# Antimalarial procurement in private-sector pharmaceutical outlets: decision-making complexities and implications for medicine quality in Tanzania

**DOI:** 10.1136/bmjgh-2022-010821

**Published:** 2023-09-10

**Authors:** Gerry Mshana, Tubeti Mayebe, Rebecca Balira, Heather Hamill, Kate Hampshire

**Affiliations:** 1 National Institute for Medical Research, Mwanza Research Centre, Mwanza, Tanzania; 2 Department of Sociology, Oxford University, Oxford, UK; 3 Department of Anthropology, Durham University, Durham, UK

**Keywords:** health systems, public health, malaria, cross-sectional survey, qualitative study

## Abstract

Poor-quality medicines are a major threat to healthcare provision in low-income countries. The problem exacerbates disease vulnerabilities of already disadvantaged populations including children, women, and the elderly. However, while the higher-level structural drivers of this problem are well established, little is known about decision-making lower down pharmaceutical supply chains, and whether this might produce vulnerabilities for medicine quality. We conducted a mixed-methods study to explore retailer–supplier interactions and decision-making dynamics for antimalarial medicines in three regions of Tanzania: Tabora, Dodoma and Mbeya. A survey questionnaire was administered to 118 small scale-and mid-range retailers in urban and rural districts of the regions. We then conducted 12 in-depth interviews with staff and owners of medicine outlets in 2 districts of Tabora region to explore further the decision-making dynamics. Results show that private-sector retailers are driven first and foremost by business and economic practicalities when choosing a medicine supplier, prioritising low purchase price, free delivery, and availability of credit. Many also rely on suppliers with whom they have personal connections, developed either within or outside the business context. Medicine quality comes far lower down the list of priorities. These findings are perhaps not surprising in a context where businesses serving low-income customers are operating on very small margins. However, when price and personal connection eclipse any other considerations, there is a risk that poor-quality medicines may find their way into supply chains, especially in countries where regulatory capacity is limited, and pharmaceutical supply chains are complex and opaque.

**WHAT IS ALREADY KNOWN ON THIS TOPIC**

- Poor-quality medicines are a major threat to healthcare provision in low-income countries. The problem exacerbates disease vulnerabilities of already disadvantaged populations including children, women and the elderly. The higher-level structural drivers of this problem are well established.

**WHAT THIS STUDY ADDS**

- Knowledge about decision-making lower down pharmaceutical supply chains. Specifically, private-sector retailers prioritised low purchase price, free delivery and availability of credit over medicine quality.
- Personal connections with suppliers were more important than considerations of medicine quality.

**HOW THIS STUDY MIGHT AFFECT RESEARCH, PRACTICE OR POLICY**

- When price and personal connection eclipse any other considerations, there is a greater risk that poor-quality medicines will penetrate supply chains, especially in countries where regulatory capacity is limited and pharmaceutical supply chains are complex and opaque.

## Introduction

Substandard and falsified (SF) medicines obstruct the provision of good healthcare and exacerbate the risk of antimicrobial drug resistance, especially in low-income contexts. The use of poor-quality medicines increases the vulnerabilities of already at-risk populations in such contexts, including children, women, and the elderly. In this paper, we use the term poor-quality medicines to include both substandard and falsified medicines as defined by the World Health Organisation (WHO).^1^ These refer to medicines which fail to meet national or international quality standards and those which are deliberately or fraudulently produced to misrepresent by their identity, composition, or source. The WHO estimates that about 10% of pharmaceutical products worldwide are of poor quality.^2^ Antimalarials are particularly vulnerable to substandard production and falsification as they are very widely used in low-income and middle-income countries (LMICs), which often have limited capacities for monitoring and regulation.^3–7^ A nationwide survey of antimalarial formulations in Tanzania found that 12% were of poor quality.^8^ Such poor-quality medicines cause therapeutic failure and drug resistance.^5 9^ Several factors contribute to the proliferation of poor-quality medicines in low-income countries like Tanzania, including antiretrovirals for the management of HIV.^10^ These include deliberate falsification and fraud (e.g., by manufacturers, suppliers and retailers); inadequate technical capacity to carry out appropriate quality assurance and post-market surveillance; weak regulatory capacities; and a high demand for affordable medicines.^8^ Private-sector supply chains are particularly prone to these vulnerabilities as business considerations may be prioritised over service provision and quality. These vulnerabilities increase in low-income contexts where the reach of public sector health facilities is limited making the majority of the population rely on private-sector retailers.^11–13^

In the context of such complexities, it is important to determine how different actors operating within pharmaceutical supply chains (especially in the private sector) make decisions around the procurement of medicines, and the implications of this decision-making for possible infiltration of poor-quality medicines. Assessing how medicine retailers of different sizes and geographical settings interact with their suppliers and make purchasing decisions is important for informing efforts to address the prevalence of poor-quality medicines. We report results from a mixed-methods study conducted in rural and urban areas of Tanzania, to explore retailer–supplier interactions and decision-making dynamics in the procurement of antimalarial medicines. We assessed the main reasons guiding the retailers’ decisions and their implications for vulnerability to SF medicines in such settings.

## Methods

### Sampling of regions and districts

This mixed-methods cross-sectional study was conducted in three purposively sampled regions of Tanzania: Tabora, Mbeya and Dodoma. The regions were sampled from three broader geographical zones of the country. Tabora, with a population of 2.2 million people and malaria prevalence of 12% for children aged under 5 years, represented the western zone. Mbeya, with a population of 2.7 million and malaria prevalence of 4% for the under 5, represented the southern highlands. And Dodoma, with a population of 2.08 million and malaria prevalence of 1% for the under 5s, represented the central zone.^14^ All districts in each selected region were stratified into urban and rural strata, and one district was randomly sampled from each category. In total, six districts were sampled (three urban and three rural). Tabora urban and Igunga were sampled from Tabora, Mbeya and Kyela from Mbeya, and Dodoma municipal and Chamwino from Dodoma.

### Sampling of facilities

We obtained a list of all registered accredited drug dispensing outlets (ADDOs) and pharmacies from the Tanzania Pharmacy Council. By the year 2020, the list had a total of 1735 pharmacies and 14 699 ADDOs. For the purpose of this study, we characterised the ADDOs as small-scale retailers and the pharmacies as mid-range retailers. The list was stratified by region and district, with each sampled district independently listed with a unique identification number. Subsequently, using a simple random sampling approach, 10 pharmacies and 10 ADDOs were selected from each district. However, in some districts, the number of pharmacies was less than 10, in which case all the pharmacies in the district were sampled and complemented with ADDOs to reach the number of 10.

### Data collection and analysis

Data collection took place in February and March 2022. A structured questionnaire was administered to one participant at each selected facility using the Census and Survey Processing system through hand-held tablets. The survey instrument consisted of four main sections: (A) location and characteristics of the outlet; (B) choice of antimalarial products and reasons; (C) choice of suppliers for antimalarial medicines and reasons and (D) regulation, with closed-response questions used throughout. The survey data were electronically transferred to servers at the National Institute for Medical Research in Mwanza and uploaded to Stata V.15.0 (STATA) for cleaning and analysis. Descriptive statistics were conducted to describe the facility characteristics and the reasons for using suppliers. Fisher’s exact test was computed to determine the difference between the background information of the ADDOs and pharmacies.

Qualitative in-depth interviews in Tabora region were conducted after completion of the survey in the three regions, to follow-up in more depth on reasons driving decisions about which supplier(s) to use. The 12 participants were purposively sampled to represent the different characteristics of the retailers in the survey, including size, geographical location and the reported number of suppliers used for antimalarials. A semi-structured guide was used with questions and follow-up probes on the specific reasons for choosing particular suppliers. An information sheet about the study aims and procedures was discussed with all participants before obtaining written (survey) and oral (in-depth interviews) consent for the interviews. Three of the authors (GM, HH and KH) jointly conducted the interviews and took detailed notes. The interviews were conducted in Swahili and English based on the participants' language abilities and preferences. Having already completed the survey questionnaire, the interviewees were familiar with the topics we wanted to discuss and the interviews had a conversational tone and style. After 2–3 interviews, the notes were compared and reviewed and the emerging themes discussed. The post-interview reviews produced extra probing questions for successive interviews. Emerging themes were identified and consolidated in the process of interviewing and reviewing of notes.

### Patient and public involvement statement

Patients or the public were not involved in the design, or conduct, or reporting, or dissemination plans of our research.

## Results

### Outlet characteristics (survey sample)

Altogether, we surveyed 118 owners (or staff representatives) of pharmacies and ADDOs in the three regions of Tanzania (table 1). Of the outlets visited, 69 (58.5%) were ADDOs and 49 (41.5%) were pharmacies. There were fewer pharmacies than ADDOs in the rural districts. For Dodoma City Council, sampled ADDOs had been upgraded to pharmacies at the time of the survey hence they had to be replaced. ADDOs named between 1 and 4 suppliers they currently used for antimalarial medicines, while pharmacies mentioned between 1 and 5, with a median of 2 suppliers for both ADDOs and pharmacies.

**Table 1:** Outlet characteristics

Facility type	ADDO (N=69)		Pharmacy (N=49)	
Variable	n	%	n	%
Region				
Dodoma	21	30.4	18	36.7
Mbeya	25	36.2	14	28.6
Tabora	23	33.3	17	34.7
District				
Dodoma city council	3	4.3	16	32.6
Chamwino	18	26.1	2	4.1
Mbeya city council	9	13.0	10	20.4
Kyela	16	23.2	4	8.2
Tabora	10	14.5	10	20.4
Igunga	13	18.8	7	14.3
Location				
Urban	22	31.9	36	73.5
Rural	47	68.1	13	26.5
No of suppliers (median, range)	2	1–4	2	1–5

ADDO, accredited drug dispensing outlet.

### Numbers of suppliers and reasons for their choice survey data

In total, the interviewed retailers listed 52 different suppliers that they were currently using to supply antimalarial medicines. There was substantial overlap of suppliers used by the retail outlets: 25 of the 52 suppliers were used by just one outlet, right up to one supplier that was mentioned by 41 different outlets. In total, we have data on 269 retailer-supplier dyads (151 ADDO supplier and 118 pharmacy supplier), shown in table 2.

**Table 2:** Reasons for using a particular supplier (denominator is number of times mentioned)

Type of facility	ADDOs (N=151)		Pharmacy (N=118)		Total (N=269)	
Reason given for using supplier	n	%	n	%	n	%
**Low price**	93	61.6	68	57.6	161	59.9
**Good customer service**	85	56.3	50	42.4	135	50.2
**Reliable/quick supplies of medicine**	41	27.2	52	44.1	93	34.6
**Geographical proximity/convenient to reach**	30	19.9	30	25.4	60	22.3
**Available on credit/good credit terms**	19	12.6	36	30.5	55	20.4
**Free delivery**	27	17.9	28	23.7	55	20.4
*Reputation for trustworthiness*	37	24.5	21	17.8	58	21.6
*Personal recommendation/connection/relationship with supplier*	18	11.9	13	11.0	31	11.5
**They have a good range of products**	10	6.6	16	13.6	26	9.7
**Always used this supplier**	16	10.6	8	6.8	24	8.9
Reputation for good-quality products	11	7.3	9	7.6	20	7.4
Just trying them out (e.g., they are a new business or have new product)	4	2.6	1	0.8	5	1.9

ADDOs, accredited drug dispensing outlets.

Table 2 lists, in descending order of frequency, the reasons given by the retailers we interviewed for choosing their particular suppliers (N.B. they could list more than one response). Most of the reasons given fall into two broad categories: business economics and other practicalities (in bold) and a personal connection or recommendation (in italics). Of these, business practicalities/economics appear to dominate. Almost 60% of retailers cited low price as a reason for using their chosen supplier(s), with good customer service, reliable/prompt supplies, geographical proximity, and credit and delivery terms all high up in the list. Personal connections and personal recommendations generally featured below business practicalities but were nonetheless important for many, and certainly appeared to be more important than reputation for good-quality products, which was one of the least-mentioned reasons for choosing a supplier (7.4%).

On the face of it, these findings may be both unsurprising and potentially alarming. They are perhaps unsurprising because, as private businesses, ADDOs and pharmacies need to stay afloat, so going for suppliers who sell medicines cheaply, who can deliver on time (and, even better, for free) and are willing to sell on credit makes good sense. But it is also alarming that so little attention is apparently being paid to medicine quality (as opposed to quality of the service).

### Affordability and personal connections: in-depth interview findings

The in-depth interviews allowed us to probe further into the decision-making process: particularly how retailers balanced different kinds of considerations (business practicalities, maintaining personal relationships and ensuring that customers received good-quality medicines) and manage any potential tensions between these. Two main themes emerged: the importance of affordability and the role of personal connections.

Interviews confirmed the paramount importance of low price for the medicine retailers. Many of those in our sample, especially ADDOs, were serving communities of low socioeconomic status, whose purchasing power was very limited. Selling medicines that their customers could afford to buy not only made business sense, it was also morally important for those who felt that they were providing an important community service.^15^ This often meant stocking the very cheapest products, even though some expressed doubts about the quality of these compared with better-known, higher-priced brands. It also sometimes meant selling medicines on credit (which may or may not ever be paid back) and even sometimes selling incomplete doses of antimalarials (and other medicines) to those who could not afford a full course (and whose credit record was too bad to risk further losses). Others reported selling cheaper antimalarials that were not licensed for the purpose intended (box 1).

Box 1 **Case study 1: the pressures of affordability**

Juma (pseudonym), an employee of a small, accredited drug dispensing outlets in Tabora, showed us the antimalarials he currently had in stock: a couple of local generic brands of ‘ALU’ (artemether–lumefantrine tablets, currently recommended by the WHO to treat acute malaria in adults and children) plus four or five generic versions of ‘SP’ (sulfadoxine–pyrimethamine, currently recommended only for intermittent prophylaxis in pregnancy, not for treating malaria). He deliberately chose suppliers who provided low-cost products; the retail price for the generic ALU was TZS3000 (c. 1 GBP) for an adult treatment, and TZS1500 for prophylactic SP. However, many of his customers could not afford even these amounts and so he had to improvise. To customers he knew well and trusted, he might agree to sell on credit, with the customer giving what they could afford and agreeing to return later with the balance. For others, with even more limited means, or those he knew from previous experience not to trust with credit, he would on occasion resort to selling a half-dose (usually on the promise that the customer would return to purchase the second half later, whether or not that actually happened) or selling the cheaper SP rather than ALU to someone suffering acute malaria. In these cases, Juma knew this was not really the ‘right’ thing to do but it was better than to leave the customer with no medicine at all.

The interview data also confirmed the importance of personal connections for some retailers and added more detail on how these work in practice. Notably, the connections were not always direct (business owner to business owner); some were mediated by employees and others, but nonetheless exercised a considerable influence on purchasing decisions. The interviews revealed two types of personal connections: relationships formed outside of business, and those formed while doing business together. In the former case, these relationships pre-dated the business relationship (rooted sometimes in extended kin networks or through having attended the same church, school or college) but then influenced and became reinforced in the course of doing business together (box 2). In the latter situation, personal connections were forged over time through the business relationship, and were reinforced through face-to-face interactions, conversations, exchange of phone contacts and ‘gifts’ from suppliers such as diaries and calendars. Although rooted in business, some retailers reported feeling a strong sense of personal connection to certain suppliers to the extent that they felt morally obliged to continue purchasing from them (box 3).

Box 2 **Case study 2: pre-existing personal connections**

Mashaka (pseudonym) is employed in a small, accredited drug dispensing outlets (ADDO) on the outskirts of Tabora, where he has worked since completing college about a year ago. He oversees purchasing medicines for the ADDO and always uses the same supplier, based in the town centre, where a former college-mate and friend of his works as an employee. Mashaka tells us that his friend can pass on up-to-date information on medicine prices and stock availability, which is very helpful. Mashaka’s allegiance to his friend runs deep: even if his first-choice products are not in stock, he told us that he would rather buy an alternative from his friend’s company (and based on his friend’s advice) than look elsewhere.

Box 3 **Case study 3: connections forged through doing business**

Asha (pseudonym) works at a small, accredited drug dispensing outlets in Tabora where she is left in charge of purchasing. As such, she interacts regularly with the local suppliers—or at least their delivery staff. They know her by name and often bring round ‘gifts’ of calendars, pens and other some items. Through these interactions over the years, Asha feels that she has come to know these suppliers personally, and ‘feels bad’ if she does not order from one of them for a while. They might also follow-up with a phone call, asking her how she is and why she isn’t buying from them. As a result, and in contrast to some others like Mashaka, Asha rotates between several local suppliers to maintain and develop this relationship.

## Discussion

This study demonstrates the decision-making complexities which private-sector retailers in urban and rural Tanzania engage in when ordering antimalarials and other medicines from suppliers. As shown, most retailers prioritise low price and other business imperatives (availability of credit, proximity to supplier, free delivery) when choosing suppliers and medicines. They do this, at least in part, because of the economic situation of their customers, many of whom can only afford the cheapest generic products, and sometimes not even appropriate doses of those. A second important set of factors appears to revolve around personal connections with suppliers: connections that may pre-date the business relationship or be formed in the process of doing business together. Both sets of factors appear to far outweigh direct considerations of medicine quality when it comes to choosing suppliers.

This situation has several potentially problematic implications for the quality of medicine reaching those in need. First, there is a risk that quality may be compromised if retailers prioritise low price over any other consideration. Although expensive medicines may also be falsified, we hypothesise that they are less prone partly due to affordability and their slow-moving nature in low-income contexts like Tanzania. Some of our interviewees clearly had doubts about the quality of some of the cheapest products they sold but knew that their customers could not afford anything else. Not enough is known about the complex relationship between price and the probability of a medicine being SF to know whether our interviewees are correct in their assessment. For example, Bate *et al* argue that relying on price as the main signal of quality carries significant risks. Somewhat counterintuitively, they find that among generics a higher price could imply a higher chance of falsification.^16 17^ But the mere fact that consumers in our study cannot afford to take quality into account in purchasing decisions is worrying. Moreover, we know that some customers cannot afford even the most basic generic antimalarials, and end up receiving partial doses, non-recommended treatments or nothing at all. Even a top-quality drug is likely to result in substandard outcomes if the wrong dose (or indeed the wrong drug) is administered.^18^

Second, while personal relationships between retailers and suppliers can help smooth the course of business (e.g., by enabling the quick flow of up-to-date information or through giving preferential terms of credit), risks may emerge when those relationships become so important that they obscure other considerations (such as quality). In both case studies 2 and 3, personal relationships were completely driving supplier decisions such that (in case 2) an alternative would not be considered even if the preferred medicine was not available, or (in case 3) suppliers were used simply to avoid causing upset to the relationship. In both of those cases, the retailers effectively entrusted the task of assuring medicine quality further up the supply chain, to suppliers whose integrity they didn’t think to question. The degree to which personal connection is important in driving decision-making can be difficult to establish. The qualitative work we did suggests that some of these influences may be quite subtle and are perhaps not picked up fully by survey methodology alone.

These findings illustrate how the conflict of interest between assurance of medicine quality and the wider economic interests play out in a lower income context like Tanzania. As shown, the economic and business interests outweighed quality assurance when the retailers chose the suppliers, contra to the main preoccupations of the end users and of the national and international medicine quality regulators. When personal interest takes precedence in a weak regulatory context like Tanzania, the wider political economic interests of the different actors prevail. This calls for heightened, innovative and effective engagement of all stakeholders in medicine supply chains—from production, distribution and retailing particularly in low-income contexts where health systems face many challenges.

The complexity of pharmaceutical supply chains in countries like Tanzania is such that no single individual or organisation has oversight of the whole.^19^ A retailer may trust their supplier because they’ve known each other from school days, but several questions arise. How do they ascertain how trustworthy their supplier is, and how much care do they take to find out? These issues may have knock on effects on the upper levels of the supply chains. In other words, while a ‘blind trust’ based on personal connection may be an effective strategy in a small, simple system where all actors know each other and may be held accountable, it is likely to be far less so in the large, complex and often opaque supply chains that are common in many LMIC settings.^20^ A weak and uncertain regulatory environment adds to this complexity, especially when retailers have to cope with uncertainties and demands on their capital from a myriad of fees charged by regulatory authorities.^2 6^

## Conclusion

Exploring the decision-making processes by those operating private-sector medicine retail outlets is very important in helping to understand how and why people end up with ineffective medicines in low-income contexts such as Tanzania. This study illustrates the complexities which medicine retailers in the private sector in urban and rural settings of the country grapple with when making decisions about ordering antimalarials from a range of suppliers. A combination of pressing economic imperatives, the importance of personal relationships and weak regulation of complex supply chains may give rise to vulnerabilities for poor-quality medicines in such contexts where quality is least considered.

This study has several limitations. The cross-sectional design limited our ability to explore decision-making over time and whether/how retailers responded to challenges with suppliers (including around medicine quality). Moreover, we have not yet been able to complete the supply-chain mapping at the higher levels (among large-scale distributors, manufacturers, importers, etc.) or to extend our ethnographic work to more regions of the country. We plan to accomplish this in future work.

## Data Availability

Data are available on reasonable request.
